# Coordinated Translocation of Presequence-Containing Precursor Proteins Across Two Mitochondrial Membranes: Knowns and Unknowns of How TOM and TIM23 Complexes Cooperate With Each Other

**DOI:** 10.3389/fphys.2021.806426

**Published:** 2022-01-06

**Authors:** Marcel G. Genge, Dejana Mokranjac

**Affiliations:** Biozentrum — Department of Cell Biology, LMU Munich, Munich, Germany

**Keywords:** mitochondria, protein translocation, presequence pathway, TOM-TIM23 contacts, precursor transfer, intermembrane space, TOM complex, TIM23 complex

## Abstract

The vast majority of mitochondrial proteins are encoded in the nuclear genome and synthesized on cytosolic ribosomes as precursor proteins with specific mitochondrial targeting signals. Mitochondrial targeting signals are very diverse, however, about 70% of mitochondrial proteins carry cleavable, N-terminal extensions called presequences. These amphipathic helices with one positively charged and one hydrophobic surface target proteins to the mitochondrial matrix with the help of the TOM and TIM23 complexes in the outer and inner membranes, respectively. Translocation of proteins across the two mitochondrial membranes does not take place independently of each other. Rather, in the intermembrane space, where the two complexes meet, components of the TOM and TIM23 complexes form an intricate network of protein–protein interactions that mediates initially transfer of presequences and then of the entire precursor proteins from the outer to the inner mitochondrial membrane. In this Mini Review, we summarize our current understanding of how the TOM and TIM23 complexes cooperate with each other and highlight some of the future challenges and unresolved questions in the field.

## Introduction

Eukaryotic cells are defined by the presence of different membrane-enclosed compartments, cell organelles, that contain specific sets of proteins and provide specific chemical milieus. The obvious advantage of the subcellular compartmentalization is that a wide variety of cellular processes can take place simultaneously under vastly different conditions. The obvious disadvantage, however, is that eukaryotic cells needed to develop very precise mechanisms that would ensure that each protein is correctly sorted to the specific organelle where it can perform its function. In general, intracellular protein sorting relies on the presence of specific targeting signals within the proteins and on the respective receptors, usually localized on the surface of the organelle, that recognize these signals (Blobel, 2000). Upon recognition of targeting signals, proteins are translocated across or inserted into the organelle membrane, usually through some form of the translocation channel and with the input of energy. Though this basic concept also applies to protein translocation into mitochondria, the complex structure of this organelle brings many additional challenges. One of them is the need to translocate the majority of its proteins across two membranes in a coordinated manner. In this Mini Review, we briefly summarize and discuss our current understanding of this process.

### The Presequence Pathway

**Figure 1:**
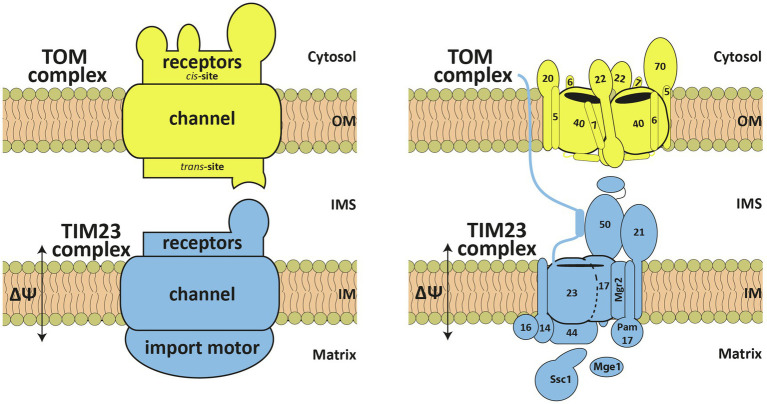
Schematic representation of the TOM and TIM23 complexes. The TOM complex consists of the receptors, Tom20, Tom22, and Tom70, and a channel unit, formed by Tom40, associated with three small proteins Tom5, Tom6 and Tom7. It possesses *cis* and *trans* presequence-binding sites. The TIM23 complex can be functionally divided into receptors, translocation channel and import motor – Tim23, Tim17, Tim50, Tim44, Tim14, Tim16, mtHsp70 (Ssc1), Mge1, Tim21, Mgr2, and Pam17. See text for details. OM, outer membrane; IMS, intermembrane space; IM, inner membrane.

Mitochondria are double-membrane-bounded organelles with four subcompartments: two membranes, the outer membrane (OM) and the inner membrane (IM), that define two aqueous subcompartments, the intermembrane space (IMS) and the innermost matrix. Though mitochondria possess their own genome, the mitochondrial DNA (mtDNA), and a complete apparatus for its expression, out of *ca*. 1,000–1,500 mitochondrial proteins, only 8 in yeast *Saccharomyces cerevisiae* and 13 in humans are encoded in the mtDNA. The vast majority of mitochondrial proteins are encoded by nuclear genes and translated on cytosolic ribosomes as precursor proteins with specific mitochondrial targeting signals. Upon initial recognition by cytosol-exposed receptors in the OM of mitochondria, precursor proteins are imported into their final place of function with the help of highly specific protein translocases present in all mitochondrial subcompartments (Neupert, 2015; Wiedemann and Pfanner, 2017; Hansen and Herrmann, 2019). The mitochondrial targeting signals are very diverse, mirroring the complex structure of the organelle. Still, about 70% of the mitochondrial precursor proteins carry at their N-termini typically 15–55 amino acids long, cleavable extensions called presequences (Vögtle et al., 2009). Presequences are characterized by the ability to form an amphipathic helix with a net positive charge (typically +3 to +6) on one side and a hydrophobic surface on the opposite side. By default, presequences target precursor proteins to the mitochondrial matrix, however, in combination with additional targeting signals, presequence-containing precursor proteins can also be targeted to the IM, IMS, and even OM. Translocation of presequence-containing precursor proteins across the two mitochondrial membranes is mediated by the TOM and TIM23 complexes in the outer and inner membranes, respectively (Figure 1). This import pathway is also termed the “presequence pathway.” As the main entry gate of mitochondria, the TOM complex is not only involved in the presequence pathway but also in import of essentially all nuclear-encoded mitochondrial proteins along other mitochondrial protein import pathways involving TOB/SAM, MIM, MIA and TIM22 complexes (Neupert, 2015; Schulz et al., 2015; Wiedemann and Pfanner, 2017; Hansen and Herrmann, 2019; Pfanner et al., 2019).

Newly synthesized mitochondrial precursor proteins are bound to cytosolic chaperones that keep them in a largely unfolded, import-competent state (Becker et al., 2019; Avendaño-Monsalve et al., 2020; Bykov et al., 2020). Presequences are recognized on the cytosolic surface of the OM, the so-called *cis* site, by the receptors of the TOM complex, Tom20 and Tom22 (Figure 1). The TOM complex has an additional receptor, Tom70. Though initial data suggested that Tom70 is specifically involved in recognition of internal targeting signals within mitochondrial proteins, recent work shows that its predominant function is in tethering cytosolic chaperones to the surface of mitochondria (Backes et al., 2021), suggesting a more general role for Tom70 in protein translocation into mitochondria. After initial recognition by Tom20 and Tom22, presequences are transferred to the translocation channel of the TOM complex formed by the β-barrel protein Tom40. Transmembrane segments of Tom22 and of three small Tom proteins, Tom5, Tom6, and Tom7, are bound on the outer surface of the Tom40 barrel. On the IMS face of the channel, presequences bind to the so-called *trans* site of the TOM complex formed by the IMS-exposed segments of Tom22, Tom40, and Tom7. Already at this stage, presequences are recognized by the IMS-exposed receptors of the TIM23 complex, Tim50 and Tim23 (Figure 1). In a membrane-potential dependent step, presequences are subsequently inserted into the still mysterious translocation channel of the TIM23 complex, formed by the membrane-embedded segments of Tim23 and Tim17. Once in the matrix, presequences are proteolytically removed by the mitochondrial processing peptidase. Translocation of the complete polypeptide chain into the matrix requires the ATP-dependent action of the import motor (Craig, 2018; Mokranjac, 2020). The peripheral membrane protein Tim44 recruits mtHsp70 (Ssc1), the ATP-consuming subunit of the motor, and its cochaperones Tim14 (Pam18), Tim16 (Pam16), and Mge1, to the translocation channel in the inner membrane. If the presequence is the only targeting signal present, precursor proteins will be completely translocated into the matrix. However, if an additional hydrophobic sorting signal (“stop-transfer signal”) is present downstream of the presequence, translocation into the matrix will be arrested and the hydrophobic segment will be inserted laterally into the IM. The TIM23 complex contains three nonessential subunits, Pam17, Tim21, and Mgr2, that appear to play a role in the differential sorting of proteins into the matrix and the IM.

It is likely that all major players of the presequence pathway are identified by now. However, molecular understanding of how presequences are recognized and handed over along this pathway is still very rudimentary – the only high-resolution structure of the receptor bound to the presequence peptide is that of the cytosolic domain of Tom20 (Abe et al., 2000; Saitoh et al., 2007). Below we present and discuss our current knowledge of how TOM and TIM23 cooperate during transfer of presequences between outer and inner mitochondrial membranes.

### Cooperation of TOM and TIM23 Complexes During Transfer of Precursor Proteins

**Figure 2:**
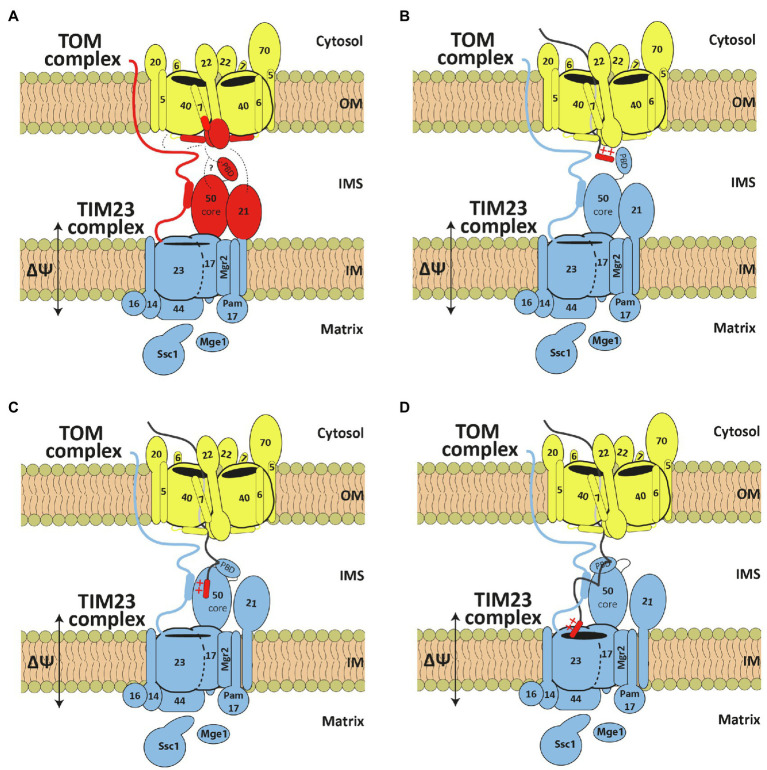
TOM and TIM23 cooperation during precursor translocation across two mitochondrial membranes. **(A)** Subunits implicated in TOM and TIM23 cooperation are highlighted in red. Dashed lines represent identified interaction points. **(B–D)** Current working model for transfer of precursors from the TOM to the TIM23 complex. See text for details. OM, outer membrane; IMS, intermembrane space; IM, inner membrane.

Upon solubilization of mitochondria, the TOM complex does not interact in a stable manner with the TIM23 complex, or with any other of the downstream translocases. Studies with isolated OM vesicles and purified and reconstituted TOM complex showed that the TOM complex is able to recognize presequence-containing precursor proteins and initiate their translocation across the OM; however, they also showed that the TOM complex on its own is not able to translocate proteins completely across the membrane (Mayer et al., 1995; Künkele et al., 1998). On the other hand, experiments performed with mitoplasts, mitochondria in which the OM was artificially removed, showed that the TIM23 complex is, on its own, able to recognize and import precursor proteins across the IM (Hwang et al., 1989). In intact mitochondria, however, N-terminal presequences can be proteolytically removed in the matrix while the C-terminal part of the protein is still in the cytosol. Also, the TOM-TIM23 supercomplex can be stabilized with precursor proteins arrested as the TOM-TIM23 spanning intermediates (Chacinska et al., 2003; Popov-Celeketić et al., 2008). These experiments show that the TOM and TIM23 complexes do not operate as isolated units but rather mediate import of presequence-containing precursor proteins in a tightly controlled and coordinated manner. The cooperation of the TOM and TIM23 complexes is likely to take place in the IMS where the two complexes meet. The subunits implicated in TOM and TIM23 cooperation are Tom22, Tom7, and Tom40, from the TOM side, and Tim50, Tim23, and Tim21, from the TIM23 side (Figure 2A).

Biochemical and genetic experiments suggested that the *trans* site of the TOM complex is formed by the IMS-facing segments of Tom22, Tom7, and Tom40. All three proteins can be crosslinked to precursor proteins arrested in the TOM complex (Kanamori et al., 1999; Esaki et al., 2004). Simultaneous deletion of Tom7 and the IMS segment of Tom22 leads to the accumulation of precursor forms of mitochondrial proteins in the cytosol, indicative of impaired import. These cells are not able to grow on a fermentable carbon source at higher temperatures and not at all on nonfermentable carbon sources (Esaki et al., 2004). The recently determined cryo-EM structure of the TOM complex indeed suggests that they are all found close to each other at the Tom40 dimer interface (Araiso et al., 2019; Tucker and Park, 2019). Unfortunately, the actual IMS-exposed segments of Tom40 and Tom22, where the presequences most likely bind, were not resolved in the structure. However, biochemical evidence has been presented that the presequence-containing precursor proteins exit the channel in the middle of the dimer where the *trans* site of the TOM complex is expected to be (Araiso et al., 2019).

Experiments performed in intact mitochondria and with isolated recombinant proteins showed that the TIM23 complex interacts with the *trans* site of the TOM complex even in the absence of protein translocation (Figure 2A). Using chemical and/or site-specific UV crosslinking, Tim23 and Tim50 were crosslinked to the IMS-exposed segments of Tom22 and Tom40 (Tamura et al., 2009; Shiota et al., 2011; Waegemann et al., 2015; Araiso et al., 2019; Günsel et al., 2020). Recombinantly expressed and purified IMS segments of Tim23 and Tim21 bound to the IMS segment of Tom22 *in vitro* (Chacinska et al., 2005; Mokranjac et al., 2005; Albrecht et al., 2006; Bajaj et al., 2014). A direct interaction of any of these TIM23 subunits with Tom7 has not been demonstrated yet, however, considering the recently shown proximity of Tom7 to the IMS-exposed segments of Tom22 and Tom40 (Araiso et al., 2019; Tucker and Park, 2019), it is likely that Tom7 is present in the vicinity of and/or interacts with at least one of the three TIM23 subunits. Indeed, Tom7 genetically interacts with the N-terminal segment of Tim23, as does the IMS segment of Tom22 (Waegemann et al., 2015). The unique feature of the N-terminal segment of Tim23 is that it is accessible to externally added proteases in intact mitochondria (Donzeau et al., 2000). Though still controversial, the exposure of Tim23 on the mitochondrial surface depends on the interaction between Tim23 and Tim50 in the IMS (Yamamoto et al., 2002; Gevorkyan-Airapetov et al., 2009; Tamura et al., 2009), the dynamics of the TOM complex (Waegemann et al., 2015), the energetic state of the inner membrane (Günsel et al., 2020) and the translocation activity of the TIM23 complex (Popov-Celeketić et al., 2008). Whether Tim23 reaches the cytosol through the lipid bilayer, through the TOM channel or by some other, still unknown mechanism remains unclear. Even though this segment of Tim23 is not essential for cell viability (Chacinska et al., 2003; Waegemann et al., 2015), it is only logical that, by crossing two mitochondrial membranes, Tim23 would bring them closer, facilitating transfer of proteins between TOM and TIM23 complexes.

The IMS-exposed segments of the TIM23 subunits not only interact with the *trans* site of the TOM complex but also with each other. The high-resolution structural information on these interactions is unfortunately still missing. Still, biochemical experiments showed that Tim21 binds to Tim23 and to Tim50, as judged by both *in organello* crosslinking and interactions between recombinantly expressed and purified proteins (Tamura et al., 2009; Lytovchenko et al., 2013; Bajaj et al., 2014). The interaction between Tim23 and Tim50 has been extensively analyzed *in vivo*, *in organello*, and *in vitro*, and residues in both proteins have been identified that contribute to their interaction (Geissler et al., 2002; Yamamoto et al., 2002; Mokranjac et al., 2003, 2009; Meinecke et al., 2006; Alder et al., 2008; Gevorkyan-Airapetov et al., 2009; Tamura et al., 2009; Qian et al., 2011; Schulz et al., 2011; Lytovchenko et al., 2013; Günsel et al., 2020; Gomkale et al., 2021). Interestingly, Tim23 seems to bind to two distinct patches on the surface of Tim50 (Tamura et al., 2009; Qian et al., 2011; Dayan et al., 2019). Also, lipids seem to play an important role in the interaction between the two proteins (Malhotra et al., 2017). Despite many efforts, the picture of Tim23-Tim50 interaction and particularly its dynamics remains blurry, likely due to the intrinsically disordered character of the Tim23 segment involved in the interaction (Gevorkyan-Airapetov et al., 2009; de La Cruz et al., 2010; Günsel et al., 2020).

Presequences are recognized by several components of the presequence pathway and precursor proteins influence not only the interactions between TOM and TIM23 complexes but also the interactions among the TIM23 subunits. Precursor proteins arrested at the *trans* site of the TOM complex can already be crosslinked to Tim50 (Yamamoto et al., 2002; Mokranjac et al., 2003, 2009), making it the first subunit of the TIM23 complex to recognize and bind presequences. The actual binding of presequences to the IMS segment of Tim50 was subsequently confirmed in a reconstituted system (Marom et al., 2011). Details of how translocating precursor proteins are transferred from the *trans* site of the TOM complex to Tim50 remain unclear. Precursor proteins increased the efficiency of chemical crosslinking between Tim50 and Tom22 (Waegemann et al., 2015), but they decreased a site-specific crosslink between the two proteins (Shiota et al., 2011). The situation got complicated even further when it was shown that the IMS segment of Tim50 consists of two domains: the highly evolutionary conserved core domain and the fungi-specific presequence-binding domain (PBD; Schulz et al., 2011). Though PBD was initially suggested to be solely responsible for recognizing and binding presequences, subsequent experiments showed that the core domain also binds presequences and even with similar affinity as PBD (Schulz et al., 2011; Lytovchenko et al., 2013). Recent NMR experiments indicate that the two domains of Tim50 bind to each other and that their interaction is modulated by presequences (Rahman et al., 2014). The receptor function of Tim50 depends on its interaction with Tim23 (Mokranjac et al., 2009; Tamura et al., 2009). Tim23 on its own also binds to presequences, however, with far lower affinity than Tim50 (Bauer et al., 1996; de La Cruz et al., 2010; Marom et al., 2011; Lytovchenko et al., 2013). Presequences dissociated the interaction of Tim50 with Tim21, indicating that Tim21 modulates the dynamic interplay of the TOM and TIM23 subunits in the IMS with presequences (Lytovchenko et al., 2013). Recent purification of the TOM-TIM23 supercomplex followed by crosslinking and mass spectrometry identified many new potential TOM-TIM23 interactions (Gomkale et al., 2021). Particularly interesting are the multiple contacts between Tim21 and Tom22 as well as the ones between Tim23 and Tom5 and Tom40. Unfortunately, these new contacts were not yet analyzed in intact mitochondria. It is also surprising that the TOM-TIM23 crosslinks previously identified in intact mitochondria were not recapitulated in this work.

Based on the available data, the current working model of how presequences are transferred from the *trans* site of the TOM complex to the translocation channel in the inner membrane would suggest the following scenario. Precursor proteins exit the TOM channel at the *trans* site where presequences are recognized by Tim50 (Figure 2B). The changes in multiple interactions between TOM and TIM23 complexes, induced by the recognition of presequences, would allow the presequences to be released from the *trans* site of the TOM complex, likely, to the PBD of Tim50. Binding of presequences to the PBD would then induce structural rearrangements within Tim50 so that presequences are further transferred to the core domain of Tim50 and the IMS-exposed segment of Tim23 (Figure 2C). In a membrane-potential dependent step, presequences are finally inserted into the TIM23 channel for translocation across the IM (Figure 2D).

## Concluding Remarks

Even *ca*. 30 years after identification of its first components, the presequence pathway still withholds many of its secrets. The major players involved in cooperation between the TOM and TIM23 complexes are probably all identified; however, we are only beginning to understand their multiple and dynamic interactions that underlie transfer of precursor proteins between two mitochondrial membranes and many open questions remain. Many of the TOM-TIM23 contacts have only been identified and their dynamics during translocation of proteins have not yet been analyzed. The different steps during transfer of presequences from the TOM to TIM23 complex remain speculative and unclear even on the level of components involved at different proposed steps. Understanding of presequence recognition in the IMS is still very limited, both on the level of the TOM and TIM23 complexes. Do the two domains of Tim50 bind presequences individually, do they together form a presequence-binding site or are they maybe involved in recognition of presequences during different stages of protein translocation? How does Tim23 contribute to presequence recognition in the IMS? Which domain of Tim50 is involved in which of the identified interactions of Tim50 and how do they change during translocation of proteins into mitochondria? If PBD of Tim50 is indeed only fungi-specific, how do higher eukaryotes deal with the lack of this domain? On a more general note, it will be interesting to know whether newly synthesized precursor proteins in the cytosol already know which TOM complexes are associated with TIM23 complexes or if the coordination of the two complexes predominantly happens after the presequences have reached the *trans* site of the TOM complex. If former, what distinguishes TOM complexes bound to TIM23 from the rest?

The successful use of the recent developments in the cryo-EM to solve the high-resolution structure of the TOM complex (Araiso et al., 2019; Tucker and Park, 2019; Wang et al., 2020) represents a milestone toward understanding the molecular mechanisms of protein import through the TOM complex and its coordination with other protein translocases. The structure of at least part of the TIM23 complex will hopefully become available soon (Sim et al., 2021). The ability to generate and purify the TOM-TIM23 supercomplex gives hope that the same developments can also be used to solve the structure of the supercomplex. The structures will certainly help in putting all the already available data in the structural context but also in raising novel hypotheses that can then be tested in careful biochemical experiments. The secrets of the presequence pathway seem less out of reach now than they were just few years ago.

## Author Contributions

MG and DM wrote the manuscript together and authors approved the submitted version. MG made the figures with the input from DM.

## Funding

This work was supported by the Deutsche Forschungs gemeinschaft (MO1944/3-1 to DM).

## Conflict of Interest

The authors declare that the research was conducted in the absence of any commercial or financial relationships that could be construed as a potential conflict of interest.

## Publisher’s Note

All claims expressed in this article are solely those of the authors and do not necessarily represent those of their affiliated organizations, or those of the publisher, the editors and the reviewers. Any product that may be evaluated in this article, or claim that may be made by its manufacturer, is not guaranteed or endorsed by the publisher.
